# Chronic illness needing palliative care in Kinshasa hospitals, Democratic Republic of the Congo (DRC)

**DOI:** 10.1186/s41182-017-0052-y

**Published:** 2017-05-05

**Authors:** Jacques Lofandjola Masumbuku, Ernest Sumaili Kiswaya, Philippe Mairiaux, Daniel Gillain, Jean Petermans

**Affiliations:** 1 Higher Institute of Medical Engineering, Kinshasa, Democratic Republic of the Congo; 2 School of Public Health, Faculty of Medicine, University of Liège, Sart-Tilman B23, 4000 Liège, Belgium; 3 Renal Unit, Faculty of Medicine, University of Kinshasa, Kinshasa, Democratic Republic of the Congo; 4 Department of Geriatrics, Faculty of Medicine, University of Liège, Liège, Belgium

**Keywords:** Palliative care, Hospitals, Kinshasa, Chronic conditions

## Abstract

**Background:**

Chronic illnesses are a major public health problem in low-income countries. In the Democratic Republic of the Congo (DRC), few data are available, especially in palliative care. In this context, the present study aimed at describing the patterns of diseases in Kinshasa hospitals as well as risk factors associated with patients’ evolving status and length of hospital stay.

**Methods:**

A prospective study was conducted in ten hospitals of Kinshasa, over a 1-year period. A total of 2699 patients with a chronic condition (non-communicable diseases (NCD) and/or AIDS) were consecutively enrolled in the study between January and December, 2013.

**Results:**

Out of 2699 patients studied, 36.9% were suffering from cardiovascular diseases, 29.7% from comorbidity and 17.5% from AIDS. 27.5% of patients died while hospitalized, and 67.4% were lost to follow-up. The risk factors independently associated with death in hospitals were AIDS (adjusted OR = 2.2) and age over 65 years old (adjusted OR = 1.7).

Peri-urban and rural areas were significantly associated with a mean adjusted hospital stay longer than 3 days. The length of stay (LOS) was shorter for women and patients living in urban areas. Patients survived for a median of 10 days (range 7–20 days).

**Conclusions:**

This study reveals the high proportion of patients suffering from advanced chronic diseases, including cardiovascular diseases, AIDS and comorbidity. It demonstrates the need for palliative care (PC) in medical practices in Kinshasa, the capital of the Democratic Republic of the Congo.

## Background

Palliative care (PC) is a medical care that improves the physical, social, psychological and spiritual quality of life of a patient and goes beyond just relieving pain. A national PC policy could help coordinate efforts and strengthen knowledge and training of caregivers in this domain for low-income countries. However, in this part of the world, PC remains historically neglected in the care continuum [1]. The World Health Assembly calls on member states to improve access to PC as an essential component of the healthcare system [2].

According to the World Health Organization (WHO) global estimates, around 54.6 million deaths occurred globally in 2011; 66% of these deaths were due to non-communicable diseases (NCD); 25% were due to communicable, maternal, perinatal and nutritional causes; and 9% were due to injuries. Among these diseases, NCD were the major one requiring PC services in addition to other chronic conditions such as AIDS and drug-resistant tuberculosis [3]. PC is not only for cancer patients but is also extended to people with AIDS, severe kidney disease or renal failure (RF), heart failure, progressive neurological diseases, end-stage lung disease and other life-limiting illnesses [4]. Indeed, PC is a multidisciplinary approach to specialized medical care for people with life-limiting, serious illnesses, jeopardizing the patient’s prognosis, regardless of life expectancy. It can be provided relatively simply and at low cost [2] in health centres and hospitals, even when resources are limited. Such care aims at improving the quality of life of patients and of their families during the course of the illness and beyond death into bereavement [2].

Quality of life of at least 100 million people would be improved if the current knowledge on PC was available for all caregivers [5]. Even if most PC is provided in high-income countries, about 80% of global needs are located in low-income countries, where two patients out of three require it [5, 6]. From a contextualizing perspective, most work and service development has been undertaken to date in a minority of countries in Eastern and Southern Africa [1].

The transition towards such approach does not seem to be implemented in French-speaking Africa yet, where 11 out of 18 countries (including the Democratic Republic of the Congo, “DRC”) do not provide any PC [7]. In this country, the number of people who might need PC, the disease conditions requiring PC and the various aspects of their needs are not known.

Previous studies performed in Kinshasa highlighted that healthcare professionals do not have enough knowledge on PC and support [8]. Needs for PC are huge in the field [9], but due to the lack of implementation in Kinshasa medical practices, the curative dimension predominates and is strongly anchored in the philosophy of care [10]. Furthermore, knowledge on medical reasons leading to PC are poor, which could explain the lack of appropriate healthcare targeting patients’ real needs.

The present study aimed at describing the pattern of chronic health problems among patients hospitalized at an advanced stage and who would need PC. Furthermore, risk factors potentially associated with the patient’s outcome and duration of hospital stay were also assessed. Finally, data on the number of people needing PC and on disease conditions needing PC needs to be estimated prior to planning service in any area.

## Methods

### Study framework

The present study was performed in the city-province of Kinshasa, in DRC. This city is subdivided into six health districts with an estimated population of about ten million people [11]. Ten hospitals were selected on the basis of the following criteria: (i) accommodation capacity (our study included more than 60% of the beds available in hospitals across Kinshasa), (ii) capacity of healthcare provided to patients with an advanced chronic condition and (iii) accessibility to services. These hospitals were grouped into three categories according to their legal status: public (*N* = 6), private (*N* = 2) and confessional (*N* = 2).

### Type of study and population

We conducted a prospective study among 2699 patients with a chronic disease condition suffering from NCD and/or AIDS and hospitalized at an advanced stage in either the department of internal medicine or surgery. These patients were affected by at least one chronic disease condition, according to the criteria established by the WHO [12], including lasting for at least 6 months and evolving generally slow and irrevocable hope of cure and/or remission. We enrolled all patients with the following illnesses, at an advanced stage: oncological diseases, cardiovascular diseases (CVD), acquired immune deficiency syndrome (AIDS), comorbidity (patient suffering from at least two chronic conditions) and other diseases of unknown aetiology. In summary, 2699 patients were included in the study, because they were hospitalized, in a chronic disease condition at an advanced stage, and suffering from one of these diseases (non-communicable (NCD) and/or AIDS), between January 1 and December 31, 2013.

### Data collection

Data were collected by trained health professionals (physicians and nurses) who recorded general information on sociodemographic details, information regarding chronic illness, comorbidities, lengths of stay (LOS) in hospital and outcomes. The investigator team regularly registered any new cases and observed the evolution of patients already enrolled. Most information was collected from patients’ medical records. When data were missing in the medical record (especially with regard to residence/zone of provenance), investigators directly asked the patient or his/her family if the patient was unable to respond. All the other files with some missing data were excluded from the study (43 files). A collection sheet was prepared in advance to collect data.

### Studied variables

The variables studied were as follows:

- Sociodemographic variables: age group (three groups: 15–40, 41–65 and >65 years old), sex (male versus female) and area of origin/residency (encompasses three categories in relation to urbanization: urban, peri-urban and rural).
- The main health problems requiring PC, including oncological diseases, CVD, AIDS, long-lasting disease of unknown aetiology (category “others”) and comorbidity. In our study, the term comorbidity was defined as patient affected by at least two diseases and was categorized into three groups: (a) oncological disease associated or not with renal failure (RF) and a CVD, (b) AIDS associated or not with CVD and a chronic respiratory disease and (c) diabetes associated with RF and a CVD.
- For each patient, evolution while under treatment was categorized as death, ongoing and lost to follow-up. The patients lost to follow-up included, on the one hand, patients for whom the family signed a discharge to return home (because they could not afford medical costs) or in search of alternative solutions (traditional remedies or therapeutic fideism in churches) and, on the other hand, patients referred to other hospitals (intermediate and tertiary) or patients who escaped from the hospital.

### Ethical consideration

The present study was approved by the Ethical Committee, School of Public Health, University of Kinshasa (number ESP/CE/084/13). Administrative and medical authorities gave their agreement as well. Informed consent of all families and some hospitalized patients was obtained when appropriate, either written or oral.

### Statistical analyses

Data were encoded in Excel™ and were analysed with STATA 12.0™ software. Continuous data were described through mean and standard deviation (SD) or median, as well as 25 and 75 percentiles (P25–P75) in addition to discrete data with proportions. Pearson chi-square test was used in the univariate analysis to compare patient’s evolution in function of sociodemographic variables and chronic disease conditions. Lengths of stay were assessed through a normal probability plot. The log transformation allowed normalizing the distribution of lengths of stay in hospitals. Student *t* test and single-factor ANOVA compared the mean length of stay in the function of patients’ evolution (lost to follow-up, deceased or still hospitalized), chronic disease conditions and sociodemographic variables. The category “others”, poorly represented (0.6%), was not taken into account for some analyses. After analysing data, two independent variables emerged, i.e. LOS and patients’ evolution. Two models were used for the multivariate analysis. The first model consisted in determining factors associated with death thanks to a backward stepwise multiple logistic regression analyses. For this model, adequacy requirements of final theoretical models were assessed through the Hosmer-Lemeshow test; the detection of outliers was verified by examining the scatter diagram of standardized residuals in the function of probabilities predicted by the models. Interaction tests happened to be not significant. Adjusted odds ratio (OR) and their confidence interval (CI) deriving from the final model were inferred. The second model allowed predicting factors affecting length of stay thanks to the backward stepwise multiple logistic regression.

For the two models, the included dependent variables were those having a *p* value less than 20% in the bivariate analysis. Results with a two-tailed *p* value <0.05 were considered to indicate a statistically significant relationship.

## Results

### Description of population

During the period of study, 2699 patients were hospitalized in PC as a result of chronic illness. Among these patients, 50.4% were male. The mean age was statistically comparable according to sex (54.3 ± 15.3 years old for men and 53.3 ± 15.6 years old for women; *p* = 0.065). Almost all patients (98.6%) were hospitalized in the department of internal medicine. The median LOS (IQR) was 10 days (range 7 to 20).

### Sociodemographic characteristics, health problems and outcomes of patients

Table 1 shows that, when considering chronic conditions, 36.9% of patients were suffering from CVD, 29.7% had a comorbidity status and 17.5% had developed AIDS. Under treatment, evolution of patients showed that 27.5% of them deceased during the study while 67.4% were lost to follow-up.

**Table 1:** Sociodemographic characteristics, health problems and outcomes of patients

Characteristics	*n* (%)	Mean ± SD
Sex		
Male	1360 (50.4)	
Age (years)		53.7 ± 15.5
Chronic conditions (*n* = 2699)		
CVD	997 (36.9)	
AIDS	471 (17.5)	
Oncology	413 (15.3)	
Others	17 (0.6)	
Comorbidity	801 (29.7)	
Evolution (*n* = 2699)		
Deceased	741 (27.5)	
Lost to follow-up	1818 (67.4)	
Ongoing hospitalization	140 (5.1)	

*n* (%) number (percentage), *SD* standard deviation, *CVD* cardiovascular diseases, *Oncology* oncological diseases, *Others* disease of unknown aetiology, *comorbidity* patient with at least two health problems

### Length of stay according to patients’ evolution

**Fig. 1:**
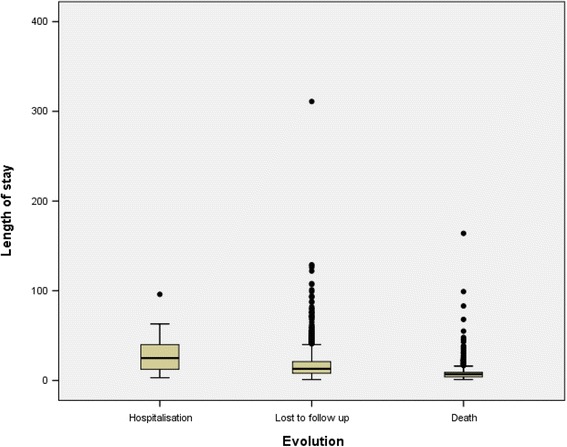
Length of stay according to patients’ evolution

The box plot of Fig. 1 shows that death occurred very early, i.e. within a median of 7 days (P75 below 10 days).

### Comorbidity categories encountered in the study population

Nearly 80% of patients suffered from CVD, renal failure and oncological disease.

### Patients’ evolution and LOS according to sociodemographic characteristics and chronic conditions

**Fig. 2:**
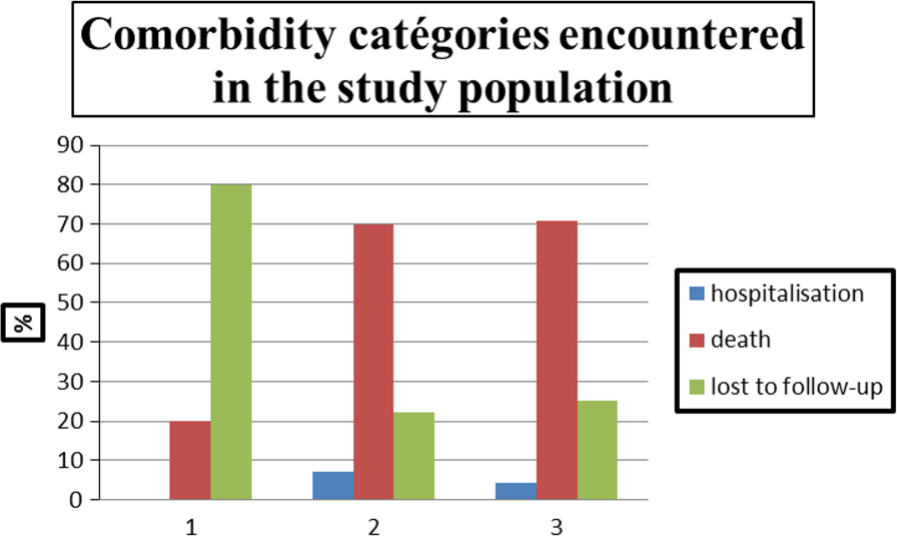
Comorbidity categories encountered in the study population. *1* oncological disease (onco) + renal failure (RF) + cardiovascular disease (CVD), *2* AIDS + CVD + chronic respiratory disease, *3* RF + diabetes + CVD

A significantly higher proportion of female respondents were hospitalized (chi-square test; *p* = 0.003). Nevertheless, proportions of lost to follow-up and deaths were very close. Nearly one third of discharged patients suffered from CVD and others from comorbidity. A slightly higher hospitalization rate was noticed in rural areas (*p* = 0.028). Considering the relationship between pathologies and outcome, we found more varied in the category of patients who remained hospitalized (*p* < 0.001) (Fig. 2).

The mean LOS was shorter for patients originating from urban areas, compared to those coming from peri-urban and rural areas (*p* < 0.001). Women remained significantly less days in hospitals (Student *t* test; *p* = 0.009) (Table 2).

**Table 2:** Patients’ evolution and length of stay according to sociodemographic characteristics and chronic conditions

Variables	Evolution			*p* value^a^	Mean length of stay (days) ±SD	*p* value^b^
	Hospi *n* (%)	Lost to follow-up *n* (%)	Death *n* (%)			
Sex				0.003		0.009
Male	52 (37.1)	943 (51.9)	365 (49.3)		16.2 ± 15.8	
Female	88 (62.9)	875 (48.1)	376 (50.7)		14.3 ± 14.9	
Age group (years)				0.432		0.234
<40	32 (22.9)	341 (18.8)	137 (18.5)		15.8 ± 14.1	
41–65	82 (58.6)	1122 (6.7)	440 (59.4)		14.8 ± 14.1	
>65	26 (18.6)	355 (19.5)	164 (22.1)		15.9 ± 19.6	
Area of origin				0.028		<0.001
Urban	23 (16.4)	403 (22.2)	159 (21.5)		12.2 ± 12.3	
Peri-urban	61 (43.6)	918 (50.5)	364 (49.1)		16.2 ± 15.6	
Rural	56 (40.0)	497 (27.3)	218 (29.4)		15.9 ± 16.9	
Chronic disease				<0.001		0.083
CVD	26 (18.7)	719 (39.8)	252 (34.2)		14.3 ± 15.9	
Oncology	40 (28.8)	262 (14.5)	111 (15.1)		16.0 ± 14.4	
AIDS	33 (23.7)	258 (14.3)	180 (24.4)		15.4 ± 17.2	
Comorbidity	40 (28.8)	567 (31.4)	194 (26.3)		15.8 ± 14.0	

*SD* standard deviation, *Hospi* hospitalization

^a^Chi-square test

^b^Student *t* test or single-factor ANOVA

### Risk factors associated with death in population studies (univariate versus multivariate analysis)

Table 3 shows that, after adjustment for sociodemographic variables and chronic diseases, two risk factors remained independently associated with death. Indeed, the risk of death was twice as high for patients suffering from AIDS and when considering patients’ age, 1.3 and 1.7 times higher for patients aged between 41 and 65 years old and more than 65 years old, respectively.

**Table 3:** Risk factors associated with death in population studied (univariate versus multivariate analysis)

Variables	Crude OR (95% CI)	*p* value	Adjusted OR (95% CI)	*p* value
Chronic disease				
CVD				
Yes	1.2 (0.9–1.2)	0.052	NA	
No	1			
Onco				
Yes	1.0 (0.8–1.1)	0.775	NA	
No	1			
AIDS				
Yes	1.8 (1.5–2.3)	<0.001	2.2 (1.7–2.7)	<0.001
No	1		1	
Comorbidity				
Yes	1.3 (1.0–1.5)	0.015	NA	
No	1			
Sex				
Male	1			
Female	1.1 (0.9–1.3)	0.470	NA	
Age group (years)				
<40	1		1	
41–65	0.9 (0.8–1.2)	0.965	1.3 (1.0–1.7)	0.027
>65	1.2 (0.9–1.5)	0.246	1.7 (1.3–2.3)	<0.001

Hosmer-Lemeshow test = 0.36, *p* = 0.948

*OR* odds ratio, *CI* confidence interval, *AIDS* acquired immunodeficiency syndrome, *Onco* oncological diseases, *NA* none applicable

### Comparison of LOS stratified by sociodemographic characteristics, outcome and chronic diseases

In this model, risk factors significantly influencing the LOS were chronic disease conditions (oncological diseases and AIDS), outcome (lost to follow-up and death), area of origin (peri-urban and rural) and being a female. Even though the variance explained by the model was low (*R* ^2^ = 0.100), after adjusting for other characteristics, the mean LOS was below 10.6 days for patients lost to follow-up and 18.3 days for deceased patients. Furthermore, AIDS and oncological diseases increased the LOS by almost 2 days. In addition, patients living in peri-urban and rural areas were associated with a mean adjusted LOS longer than 3 days, compared to patients coming from urban areas. The female sex had a higher impact on the mean LOS (Table 4).

**Table 4:** Comparison of LOS stratified by sociodemographic characteristics, outcome and chronic diseases

	*β*	SE (b)	*p* value
Length of stay (*n* = 2.699; *R* ^2^ = 0.100)			
Lost to follow-up (Y/N)	−10.604	1.124	<0.001
Death (Y/N)	−18.34	1.175	<0.001
Peri-urban area (Y/N)	3.927	0.724	<0.001
Rural area (Y/N)	3.532	0.802	<0.001
Oncological disease (Y/N)	1.541	0.747	0.039
AIDS (Y/N)	1.834	0.6	<0.009
Sex (female/male)	−1.979	0.49	<0.001

*Y* yes, *N* no, *AIDS* acquired immunodeficiency syndrome, *β* coefficient, *SE (b)* standard error, *R* ^*2*^ determination coefficient

## Discussion

The present study described the typology of health problems among patients hospitalized in PC. The analysis also highlighted factors associated with the patient evolution and length of stay. Over three quarters of patients were suffering from NCD diseases and less than one person in five was affected by AIDS. Our results confirm the findings of Krzesinski and Oni [13, 14], who showed that Africa is facing a double challenge: apart from infectious diseases, a major increase in NCD diseases must be taken into account. More than one third of patients were suffering from CVD. Damourou in Togo showed similar results [15]. Hence, advanced CVD are frequent in hospitals and affect the poorest patients and those aged between 30 and 60 years old. Some studies performed in Africa, and focusing on patients with a chronic disease condition, showed that the management of cardiovascular emergencies requires particular PC in hospitals owing to their frequency and severity [16]. It is crucial to refer patients to care on the basis of their status and to adapt their care during hospitalization.

Like Lewington et al. in Uganda [17], we found that more than one in ten people were suffering from oncological diseases. However, the prevalence of these diseases is higher compared to similar European studies. This discrepancy of prevalence between these studies could be due to the difference in methodology applied in each survey. It could also reflect the situation of our patients who are referred late at an advanced stage and had limited access to curative therapies, thus claiming for the development of adapted PC to answer to the needs of these patients [17]. The fastest increase in oncological diseases is foreseen in Africa, where near 70% of deaths occurred secondary to oncological disease in 2012 [18, 19]. Such increase in oncological diseases is responsible for a high lethality and an urgent and increased demand in PC [20, 21].

Our findings also showed that more than one in ten people were infected by AIDS. These results observed in Kinshasa hospitals approximate the observations yielded in Kenya and Uganda, where AIDS patients were diagnosed at a palliative stage [17, 22]. Indeed, most of their patients were stigmatized due to the release of their status, financial difficulties, education level and other factors [23–25]. For these reasons, they arrive late at the hospital and at a stage requiring PC.

Our observations are consistent with previous studies regarding the high frequency of patients presenting comorbidities [13, 26–34]. Literature data on comorbidity are mixed. Several authors expected that, in 2020, the burden of CVD and diabetes would increase by 130% in Africa, with a simultaneous increased incidence of chronic and end-stage renal disease (ESRD). In DRC, proofs of such trends already exist. Indeed, glomerulonephritis (35%), hypertension (30%) and diabetes mellitus (25%) all causes of ESRD currently reached levels comparable to rich countries. Most of these diseases are diagnosed at a late stage, when they are no longer reversible [13]. Some patients with comorbidity were suffering from AIDS and cancer [35], while others were seen with RF due to hypertension, diabetes and AIDS [27, 29, 30, 32–34]. In our study, more than half of patients were suffering from RF, diabetes and CVD, confirming the previous findings of Sumaili and Krzesinski who showed that three quarters of renal patients treated in Kinshasa hospitals were admitted to the hospital at an end stage [13, 30]. Given the limited renal replacement therapy (RRT) in this country, these alarming reports demonstrate the need for the implementation of other approaches such as PC.

In the present study, patients were suffering from CVD, comorbidity, oncological diseases and AIDS. Our results are consistent with the studies carried out in sub-Saharan countries by Harding and Collins [22, 35–37]. However, Lewington et al. [17] reported that patients were more affected by AIDS and oncological diseases, in addition to cardiac, renal and liver diseases. Palliative care is rarely accessible in rural sub-Saharan Africa, where more patients suffering from oncological diseases and AIDS are actually living [38]. We showed that patients who had developed AIDS were twice as likely to die. Depending on the age, the risk is respectively 1.3 and 1.7 times higher for patients aged between 41 and 65 years old and those over 65 years old. Our results corroborate previous findings [39–45]. AIDS patients remain on the sidelines of health structures because of stigmatization. They go to the hospital at an advanced and severe stage of illness and, above all, during the least opportune days of a therapeutic itinerary. Similarly, while in economic resiliency, old patients are brought to the hospital at a late stage.

The type of care provided does not seem to be of quality. Several reasons could explain such a situation, e.g. the lack of perceptions and awareness on these diseases, economic resiliency, wrong diagnosis and poor quality of care. In DRC, 87.7% of the population live below the poverty line, which is fixed at 1.25 USD per day, and 74% live below the multi-dimensional poverty line that considers access to healthcare, health and food [46]. In addition, sociocultural barriers could also explain the lack of comprehension of good care compliance [47, 48]. An advanced chronic disease is considered as an “evil spell” or “damnation”. For the management of these patients, new specific strategies of care should be encouraged. It is crucial to remain realistic towards the prognosis, to be able to discuss with the patient and his/her family and to determine the patients’ wishes regarding the location of PC while waiting for death [49].

Our results confirm the findings of Ogah et al. in Nigeria [50], who reported that more than two thirds of patients were lost to follow-up. Several reasons can explain why some patients leave the hospital, e.g. family discharge, escape from hospital, reference towards intermediary and tertiary hospitals and use of traditional medicine and therapeutic fideism (church). In our context, the team of investigation had no information on when the patients died.

Our results also showed that more than two thirds of patients with comorbidity suffered from CVD, renal failure and oncological diseases. Similar comorbidities were highlighted in previous studies undertaken in the DRC [13, 30]. In addition, the present study shows that one third of lost to follow-up patients had CVD and comorbidity. Nonetheless, the results are similar to those reported by several other authors in recent studies in the DRC [13, 26, 32, 34]. A higher proportion of hospitalized women were encountered in our study. These findings are quite similar to those reported by other African scientists [51, 52]. We also noticed that the LOS was shorter in women. Tshamba et al. [53] yielded similar result in their survey performed in Lubumbashi, another city of DRC. The reason of this discrepancy between sex remains unclear but may be attributed to the fact that men are less likely to present earlier in the disease as part of deficient health-seeking behaviour.

When considering the LOS with other associated risk factors, it appeared that patients from peri-urban and rural were hospitalized for a longer period (mean adjusted stay above 3 days). Such longer stays are also found among PC patients in other parts of Africa [54]. Our study showed that patients from urban areas remained hospitalized for less time, as already reported by Labhardt et al. in Switzerland [52] and Rosenthal in Malawi [55]. Mean shorter LOS for patients from urban areas could be for those who can be seen earlier, with more health services available, and for those who can afford them (if there is a charge) along with transport costs, compared to patients from peri-urban and rural settings. We also observed that AIDS and oncological patients had a 2-day-longer stay than other patients. An increased quality of care could have a significant impact for patients during this time. The multiple logistic regression analyses confirmed the results of the univariate analysis. The palliative approach seems to be the main component of adapted care strategies. An efficient care relying on PC could improve the quality of life and thus extend the life of patients [56]. Contextualizing PC according to our culture appears to be a new priority and requires new practices, if one wants to alleviate the suffering and efficiently care for these patients, according to their status and typology of diseases, in any healthcare facility.

### Study limitations

Our study has some limitations: this is a prospective study on hospitalized patients with chronic disease located in urban (Kinshasa) as well as in predominantly rural country. Medical records were the main source of data (for the diagnoses of chronic illness and comorbidities), which may have underestimated the need for potential palliative care if such need was poorly recorded. In addition, this study did not use the screening PC tools that have been standardized and validated in Africa. Extrapolation of the data must therefore be done with great caution. In addition, accounting for all healthcare environments in our study, including home care, would improve more our results.

## Conclusions

The study confirms that patients with a chronic disease condition are frequent in Kinshasa hospitals. They often referred late to the hospital once they have reached an advanced stage of illness. The most frequent diseases encountered were CVD, comorbidities and AIDS. One third of patients died at the hospital, and more than two thirds of patients were lost to follow-up. The proportion of female respondents being hospitalized was higher; their hospital stay was shorter, identical to patients from urban areas. Additional studies are urgently required in different environments of care in order to develop a more detailed inventory of these diseases. These will help implement appropriate healthcare strategies in DRC.

## Abbreviations

AIDS: Acquired immune deficiency syndrome
CI: Confidence interval
CVD: Cardiovascular diseases
DRC: Democratic Republic of the Congo
ESP: Ecole de Santé Publique (in French)
ESRD: End-stage renal disease
IQR: Interquartile range
LOS: Length of stay
*N*: Number
*n* (%): Number (percentage)
NCD: Non-communicable diseases
OR: Odds ratio
P: Percentile
PC: Palliative care
*R*^2^: Coefficient of determination
RF: Renal failure
SD: Standard deviation
WHO: World Health Organization
Y: Yes

## References

[1] World Health Organization (WHO) (2013). Push for palliative care stokes debate. Bull World Health Organ.

[2] World Health Organization (WHO) (2015). Palliative care.

[3] Connor SR, Sepulveda MC (2014). Global atlas of palliative care at the end of life.

[4] Vicky Lavy CB, Wooldridge R. Palliative care toolkit improving care from the roots up in resource-limited settings. http://integratepc.org/wp-content/uploads/2012/12/Pall-Care-Toolkit-Training-manual-FINAL.pdf. Accessed 31 Mar 2017.

[5] Stjernsward J, Foley KM, Ferris FD (2007). The public health strategy for palliative care. J Pain Symptom Manage.

[6] World Health Organization (WHO) (2015). First ever global atlas identifies unmet need for palliative care.

[7] Mwangi-Powell F, Dix O (2011). Palliative care in Africa: an overview, Africa Health.

[8] Lofandjola Masumbuku J, Coppieters Y (2013). Analyse des connaissances des infirmiers sur les soins palliatifs et d’accompagnement à Kinshasa, RDC. [in French]. Méd Palliat Soins Support-Accompagnement Ethique.

[9] Stjernsward J, Foley KM, Ferris FD (2007). Palliative care: the public health strategy. J Public Health Policy.

[10] Mashinda K. Prise en charge des malades en phase terminale de cancer et autres pathologies chroniques: insuffisances et pistes de solution dans l’amélioration de la qualité de vie des personnes mourantes. [in French] Thèse doctorale en santé publique. Université de Kinshasa. 2012: 145.

[11] Institut National de la statistique RDC. Annuaire statistique 2014. [in French]. 2011. Available: http://www.ins-rdc.org/sites/default/files/Montage%20AnnuStat%20FINAL%202%20From%20VEROUILLE%20_0.pdf.

[12] World Health Organization (WHO) (2013). Noncommunicable disease.

[13] Krzesinski JM, Sumaili EK, Cohen E (2007). How to tackle the avalanche of chronic kidney disease in sub-Saharan Africa: the situation in the Democratic Republic of Congo as an example. Nephrol Dial Transplant.

[14] Oni T, Youngblood E, Boulie A, McGrath N, Wilkinson RJ, Levitt NS (2015). Patterns of HIV, TB, and non-communicable disease multi-morbidity in peri-urban South Africa—a cross sectional study. BMC Infect Dis.

[15] Damorou F, Pessinaba S, Lawson B, Abdoulaye S, Soussou B, Grunitzky K (2008). [Cardiovascular emergencies and their morbimortality at the hospital. Report of 733 cases at the CHU campus in Lome (national reference hospital in Togo)]. Mali Med.

[16] Bertrand E, Muna WF, Diouf SM, Ekra A, Kane A, Kingue S (2006). [Cardiovascular emergencies in Subsaharan Africa]. Arch Mal Coeur Vaiss.

[17] Lewington J, Namukwaya E, Limoges J, Leng M, Harding R (2012). Provision of palliative care for life-limiting disease in a low income country national hospital setting: how much is needed?. BMJ Support Palliat Care.

[18] World Health Organization (WHO) (2015). Cancer.

[19] Didkowska J, Wojciechowska U, Manczuk M, Lobaszewski J (2016). Lung cancer epidemiology: contemporary and future challenges worldwide. Ann Transl Med.

[20] Rawlinson F, Gwyther L, Kiyange F, Luyirika E, Meiring M, Downing J (2014). The current situation in education and training of health-care professionals across Africa to optimise the delivery of palliative care for cancer patients. Ecancermedicalscience.

[21] Busolo DS, Woodgate RL. Using a supportive care framework to understand and improve palliative care among cancer patients in Africa. Palliat Support Care. 2015:1–18. 10.1017/S1478951515000796 26073264

[22] Harding R, Powell RA, Namisango E, Merriman A, Gikaara N, Ali Z (2014). Palliative care-related self-report problems among cancer patients in East Africa: a two-country study. Support Care Cancer.

[23] Ankrah DN, Koster ES, Mabtel-Teeuwisse AK, Arhinful DK, Agyepong IA, Lartey M (2016). Facilitators and barriers to antiretroviral therapy adherence among adolescents in Ghana. Patient Prefer Adherence.

[24] Adedigba MA, Adekanmbi VT, Asa S, Fakande I (2016). Pattern of utilisation of dental health care among HIV-positive adult Nigerians. Oral Health Prev Dent.

[25] Russell S, Zalwango F, Namukwaya S, Katongole J, Muhumuza R, Nalugya R (2016). Antiretroviral therapy and changing patterns of HIV stigmatisation in Entebbe, Uganda. Sociol Health Illn.

[26] Bukabau JB, Makulo JR, Pakasa NM, Cohen EP, Lepira FB, Kayembe PK (2012). Chronic kidney disease among high school students of Kinshasa. BMC Nephrol.

[27] Pakasa NM, Sumaili EK (2012). [Pathological peculiarities of chronic kidney disease in patient from sub-Saharan Africa. Review of data from the Democratic Republic of the Congo]. Ann Pathol.

[28] Ekulu PM, Nseka NM, Aloni MN, Gini JL, Makulo JR, Lepira FB (2012). [Prevalence of proteinuria and its association with HIV/AIDS in Congolese children living in Kinshasa, Democratic Republic of Congo]. Nephrol Ther.

[29] Makulo RJ, Nseka NM, Jadoul M, Mvitu M, Muyer MT, Kimenyembo W (2010). [Albuminuria during the screening for diabetes in a semi-rural area (Kisantu City, DR Congo)]. Nephrol Ther.

[30] Sumaili EK, Krzesinski JM, Cohen EP, Nseka NM (2010). [Epidemiology of chronic kidney disease in the Democratic Republic of Congo: review of cross-sectional studies from Kinshasa, the capital]. Nephrol Ther.

[31] Sumaili EK, Cohen EP (2010). Screening for chronic kidney disease in sub-Saharan Africa. Lancet.

[32] Sumaili EK, Krzesinski JM, Zinga CV, Cohen EP, Delanaye P, Munyanga SM (2009). Prevalence of chronic kidney disease in Kinshasa: results of a pilot study from the Democratic Republic of Congo. Nephrol Dial Transplant.

[33] Sumaili EK, Cohen EP, Zinga CV, Krzsinski JM, Pakasa NM (2009). High prevalence of undiagnosed chronic kidney disease among at-risk population in Kinshasa, the Democratic Republic of Congo. BMC Nephrol.

[34] Longo AL, Lepira FB, Sumaili EK, Makulo JR, Mukumbi H, Bukabau JB (2012). Prevalence of low estimated glomerular filtration rate, proteinuria, and associated risk factors among HIV-infected black patients using Cockroft-Gault and modification of diet in renal disease study equations. J Acquir Immune Defic Syndr.

[35] Harding R, Selman L, Agupio G, Dinat N, Downing J, Gwyther L (2011). The prevalence and burden of symptoms amongst cancer patients attending palliative care in two African countries. Eur J Cancer.

[36] Harding R, Selman L, Agupio G, Dinat N, Downing J, Gwyther I (2012). Prevalence, burden, and correlates of physical and psychological symptoms among HIV palliative care patients in sub-Saharan Africa: an international multicenter study. J Pain Symptom Manage.

[37] Collins K, Harding R (2007). Improving HIV management in sub-Saharan Africa: how much palliative care is needed?. AIDS Care.

[38] Herce ME, Elmore SN, Kalanga N, Keck JW, Wroe EB, Phiri A (2014). Assessing and responding to palliative care needs in rural sub-Saharan Africa: results from a model intervention and situation analysis in Malawi. PLoS One.

[39] Dryden-Peterson S, Medhin H, Kebabonye-Pusoebtsi M, Seage GR, Suneja G, Kayembe MK (2015). Cancer incidence following expansion of HIV treatment in Botswana. PLoS One.

[40] Hachfeld A, Ledergerber B, Darling K, Weber R, Calmy A, Battegay M (2015). Reasons for late presentation to HIV care in Switzerland. J Int AIDS Soc.

[41] Misganaw A, Mariam DH, Araya T, Ayele K. Patterns of mortality in public and private hospitals of Addis Ababa, Ethiopia. BMC Public Health. 2012;12:1007. 10.1186/1471-2458-12-1007 PMC3520706 23167315

[42] Semaille C, Lot F (2006). [Epidemiology of HIV infection in the world and in France]. Rev Prat.

[43] World Health Organization (WHO). HIV/AIDS. Available: http://www.who.int/mediacentre/factsheets/fs360/fr/. Accessed 18 Oct 2016.

[44] Arodiwe EB,Nmokedluko SC, Ike SO, Ulasi II, Ijoma CK. Medical causes of death among the elderly in a tertiary hospital, Southeast Nigeria. West Indian Med J. 2015. doi: 10.7727/wimj.2014.326. [Epub ahead of print]PMID: 27398607.

[45] Ramilitiana B, Ranivoharisoa EM, Dodo M, Razafimandimby E, Rabdriamarotia WF (2016). [A retrospective study on the incidence of chronic renal failure in the Department of Internal Medicine and Nephrology at University Hospital of Antananarivo (the capital city of Madagascar)]. Pan Afr Med J.

[46] Programme des nations unies pour le développement (2014). Revue de presse. [in French].

[47] Bain LE, Awah PK, Geraldine N, Kindong NP, Sigal Y, Bernard N (2013). Malnutrition in Sub-Saharan Africa: burden, causes and prospects. Pan Afr Med J.

[48] Schaetti C, Ali SM, Hutubessy R, Khatib AM, Chaignat CL, Weisa MG (2012). Social and cultural determinants of oral cholera vaccine uptake in Zanzibar. Hum Vaccin Immunother.

[49] Brown EA (2012). Quality of life at end of life. J Ren Care.

[50] Ogah OS, Stewart S, Falase AO, Akinyemi JO, Adegbite GD, Alabi AA (2014). Short-term outcomes after hospital discharge in patients admitted with heart failure in Abeokuta, Nigeria: data from the Abeokuta Heart Failure Registry. Cardiovasc J Afr.

[51] Dimi S, Albucher D, Zucman D (2014). Future prospectives of sub-Saharan women living with HIV residing in France for more than seven years: prospective pilot study. J Int AIDS Soc.

[52] Labhardt ND, Cheleboi M, Faturyiele O, Motlatsi MM, Pfeiffer K, Lejone TI (2014). Higher rates of metabolic syndrome among women taking zidovudine as compared to tenofovir in rural Africa: preliminary data from the CART-1 study. J Int AIDS Soc.

[53] Tshamba HM, Van Caillie D, Nawej FN, Kapend FM, Kaj FM, Yav GD (2014). Risk of death and the economic accessibility at the dialysis therapy for the renal insufficient patients in Lubumbashi city, Democratic Republic of Congo. Pan Afr Med J.

[54] Desalu OO, Wahab KW, Fawale B, Olarenwaju TO, Busari OA, Adekoya OA (2011). A review of stroke admissions at a tertiary hospital in rural Southwestern Nigeria. Ann Afr Med.

[55] Rosenthal A (2016). “Doing the Best We Can”: providing care in a Malawian antiretroviral clinic. Med Anthropol.

[56] Desrosiers T, Cupido C, Pitoul E, Van Niekerl L, Badri M, Gwyther L (2014). A hospital-based palliative care service for patients with advanced organ failure in sub-Saharan Africa reduces admissions and increases home death rates. J Pain Symptom Manage.

